# Evaluation of Six Patients with Pulmonary Carcinosarcoma with a Literature Review

**DOI:** 10.1100/2012/167317

**Published:** 2012-04-24

**Authors:** Sinem Nedime Sökücü, Celalettin Kocatürk, Nur Ürer, Yaşar Sönmezoğlu, Levent Dalar, Levent Karasulu, Sedat Altın, Mehmet Ali Bedirhan

**Affiliations:** ^1^Department of Pulmonology, Yedikule Chest Disease and Thoracic Surgery Training and Research Hospital, Zeytinburnu, 34760 İstanbul, Turkey; ^2^Department of Thoracic Surgery, Yedikule Chest Disease and Thoracic Surgery Training and Research Hospital, Zeytinburnu, 34760 İstanbul, Turkey; ^3^Department of Pathology, Yedikule Chest Disease and Thoracic Surgery Training and Research Hospital, Zeytinburnu, 34760 İstanbul, Turkey

## Abstract

*Background*. Carcinosarcoma of the lung is a rare malignant neoplasm. We evaluated the diagnosis and treatment of six carcinosarcoma cases, including a synchronous tumour and a solitary pulmonary tumour, along with the clinical and histological features and survival times. *Methods*. From a retrospective analysis of 1076 non-small-cell lung cancer resections performed between January 1996 and January 2011, six patients (0.5%) with pulmonary carcinosarcoma (all males; mean age 58 years; range 53–66) who underwent surgical treatment were studied. *Results*. The mean tumour pathological T diameter was 7.2 cm (median 6 cm, range 3–14.5 cm). Only one patient was diagnosed with carcinosarcoma preoperatively. The clinical presentation and tumour localisations differed. The operations performed were a lobectomy (*n* = 4), pneumonectomy (*n* = 1), and bilobectomy (*n* = 1). Histologically, the epithelial characteristics of the tumours were consistent with squamous cell carcinoma in most of the patients. A complete resection was performed in all six patients. No mortality occurred in the early postoperative period. The median survival time was 9 (3–25) months. *Conclusion*. The preoperative diagnosis of carcinosarcoma of the lung is difficult due to the composition of the different histopathological structures. Complete surgical resection is the treatment of choice for pulmonary carcinosarcoma, although further studies are needed.

## 1. Introduction

Pulmonary carcinosarcoma (PCS) is a rare tumour in humans [1, 2]. It was first defined by Kika et al. in 1908 as a poorly differentiated non-small-cell carcinoma containing a component with sarcoma or sarcoma-like features [3]. It accounts for 0.3 to 1% of all pulmonary cancers, and its clinical characteristics, preoperative diagnostic methods, and prognostic factors are still not completely understood [4]. Pulmonary carcinosarcomas occur predominantly in elderly men and middle-aged smokers [5]. This study evaluated the results of six cases who underwent surgery for PCS.

## 2. Material and Method

This was a retrospective study of six patients who underwent surgery for pulmonary tumours in our chest surgery clinic between January 1996 and January 2011 and who had a postoperative diagnosis of PCS. The patients were evaluated in terms of their age, gender, symptoms, diagnostic approaches, surgical methods, and followup findings.

All of the patients underwent routine laboratory studies, respiratory function tests, electrocardiography, chest X-ray, computed tomography (CT) of the thorax, brain CT or magnetic resonance imaging (MRI), abdominal ultrasound, and bronchoscopy. Three patients underwent positron emission tomography (PET)-CT. In the preoperative period, one patient without a cancer diagnosis was suspected of having lung cancer, and one patient had a diagnosis of carcinosarcoma, whereas the other four patients were diagnosed with non-small-cell lung cancer (NSCLC). The patient who had a preoperative diagnosis of carcinosarcoma had an endobronchial component and was diagnosed by bronchoscopy. The mediastinal lymph nodes were evaluated by mediastinoscopy in all but one patient. A standard posterolateral thoracotomy incision and intraoperative staging were done in all patients. Complete resection was done in all patients. All of the tumours were staged postoperatively according to the seventh international TNM staging system. The diagnosis was verified immunohistochemically.

All patients underwent clinical and radiological follow-up for a median of 7.5 (range 3–25) months. All patients were assessed quarterly for the first 2 years with a history, physical examination, and chest X-ray. Laboratory tests and advanced radiological methods were requested if there were any symptoms. Additionally, all patients or the families were asked by phone when the study was performed and asked about any signs of recurrence or complications.

The statistical analysis was done using SPSS ver. 11.5. Descriptive analyses and Kaplan-Meier survival analysis were used.

## 3. Results

All six patients were male and heavy smokers (mean 52 packs/year (median 45, range 30–90)), with a median age of 56 (range 53–66) years. The most common presenting symptom was cough (Table 1).

**Figure 1:**
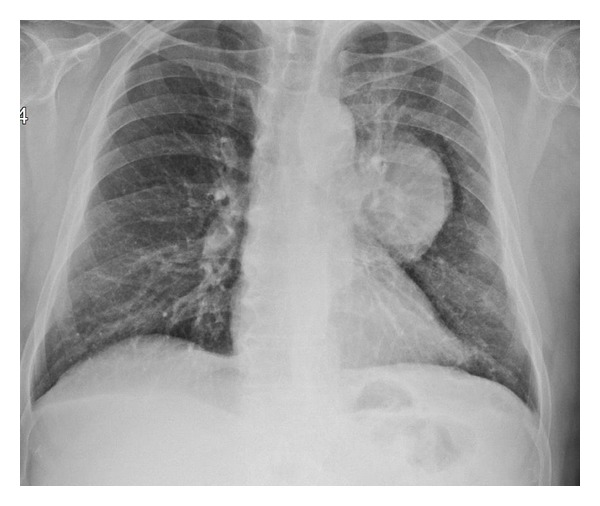
Chest X-ray of a patient showing a mass lesion located left hiler localization.

Two of the tumours were located peripherally, three were located centrally (Figure 1), and the sixth had components in both areas. The diagnosis was made by fine-needle aspiration biopsy in two patients and by fibre optic bronchoscopy in three patients. Three of the six patients underwent PET-CT. In these patients, pathological uptake was detected in the tumours, but no mediastinal involvement was detected. The operations performed were a pneumonectomy (due to fissure invasion) in one patient, bilobectomy in another, and lobectomy in the others (Table 2).

**Figure 2:**
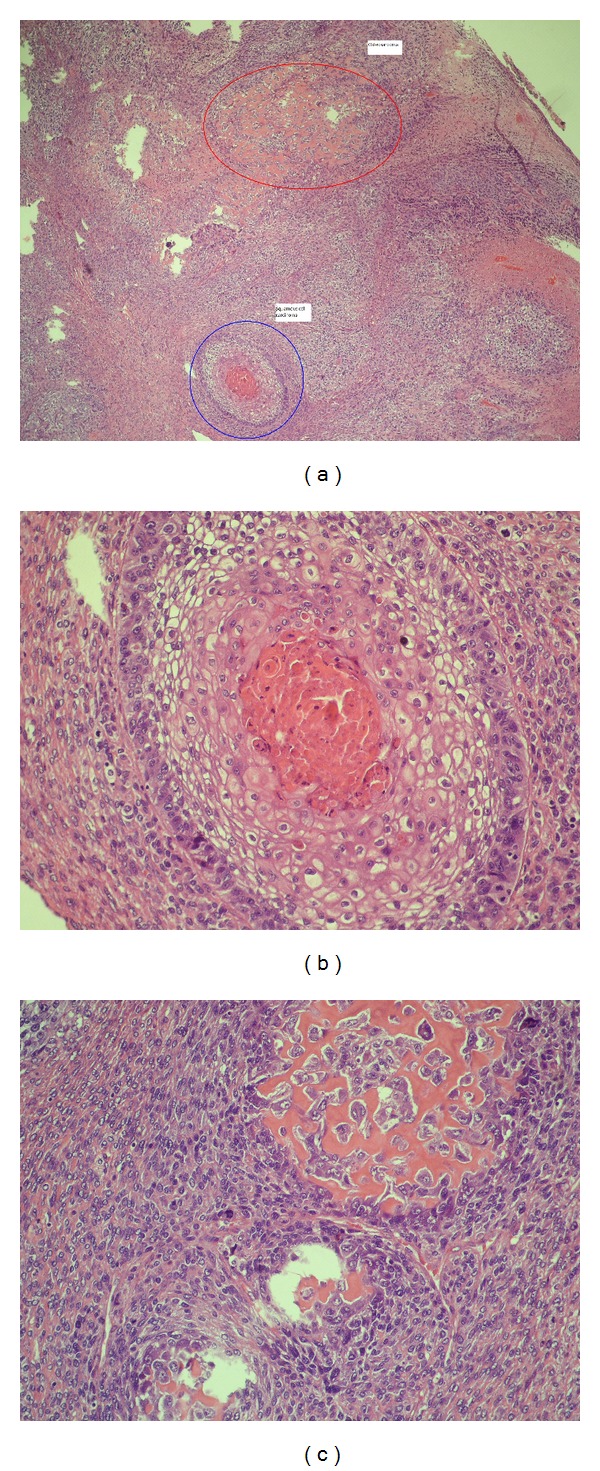
(a) Showing both squamous cell carcinoma and osteosarcoma seen in the same pies (×40 H&E). (b) With bigger magnification squamous cell carcinoma (×400  H&E). (c) Osteosarcoma components from the same pies (×400 H&E).

Histologically, the epithelial characteristics of the tumours were consistent with squamous cell carcinoma (*n* = 4) and adenocarcinoma (*n* = 2). The sarcomatous component was mostly chondrosarcomatous type and osteosarcomatous type (Table 2, Figure 2). No mortality occurred in the early postoperative period, although a bronchopleural fistula (BPF) developed in two patients in the first postoperative month. A right lower bilobectomy was done in one of these patients, and the BPF was treated with a complementary pneumonectomy and omentoplasty. In the other patient, a right pneumonectomy was done to treat the tumour invasion of the fissure, and the BPF was treated with an omentoplasty.

All of the patients were sent for oncological evaluations, and two patients with N1 disease received chemotherapy; chemotherapy was not deemed necessary for the others. In one patient, a synchronous squamous cell carcinoma (stage IA) was also detected. This patient refused further treatment after one dose of chemotherapy.

One of the patients died from brain metastasis at 10 months, and three other patients died of local metastases at 3, 5, and 9 months, respectively. Only two of the six patients were alive at the time of evaluation. The median survival time was 9 months (95% CI 1,16–16,84; range 3–25).

## 4. Discussion

Pulmonary carcinosarcoma is a subgroup of pulmonary sarcomatoid carcinomas. Currently, pulmonary sarcomatoid carcinomas are defined as poorly differentiated non-small-cell carcinomas containing a component with sarcoma or sarcoma-like (spindle or giant cell) features. Its definition was ambiguous until the recent establishment of World Health Organisation (WHO) criteria, which classify it into carcinosarcoma, pleomorphic carcinoma, and spindle cell carcinoma [6].

Pulmonary carcinosarcoma is more common among males with a smoking history. The literature reports a 4- to 7.25-fold male dominance [7–9]. In our group, all of the patients were male, with a mean age of 58 years, and all were heavy cigarette smokers, as expected.

The gross pathology of the tumour was either an intraparenchymal or intrabronchial polypoid mass. Koss et al. reported central localisation in 62% of cases [5]. The clinical presentation of the centrally localised type involves a cough, dyspnoea, and haemoptysis, like other endobronchial tumours. The second type of PCS, the peripheral solid parenchymal type, often presents as a large mass. These tumours are asymptomatic in the early stage, during which time they may involve the adjacent organs or structures such as the mediastinum, pleura, and thoracic wall [10]. Moore reported that one-third of these tumours were located peripherally [11], whereas Yazıcı et al. reported a peripheral location in 85.7% [12]. In our series, 66% of the patients had peripheral tumours, and their symptoms were consistent with the literature. Interestingly, one presented with a solitary pulmonary nodule. Although the reported radiological properties of these tumours in the English literature do not include solitary pulmonary nodule, this can also be a presentation of PCS.

In three of the six patients, PET-CT was performed. Two patients had positive uptake, and the other case was borderline. Only one paper about mean positron emission tomography uptake was published in the English language literature and it found that uptake of sarcomatoid carcinoma is significantly higher than in other types of lung cancer (*P* < 0.0001) [13]. No other information on the PET-CT findings of PCS of the lung has been reported, and the findings in our series were variable.

In our cases, the mean tumour diameter was 7.2 cm, which concurs with the literature. Our series had lower-lobe dominance in contrast to the reported upper-lobe dominance [5, 10, 11].

Immunohistochemical techniques are used to differentiate the epithelial and mesenchymal tumour elements of carcinosarcomas. The carcinomatous component is non-small-cell lung carcinoma, including squamous cell carcinoma, adenocarcinoma, and large-cell carcinoma. The most common epithelial component is squamous cell carcinoma. Takeda et al. reported a rate of 69% for squamous cell carcinoma, 24% for adenocarcinoma, and 6% for a combination of carcinomas [8]. Koss et al. reported that the most frequent epithelial component was squamous cell carcinoma (46%), followed by adenocarcinomas (31%) and adenosquamous cell carcinoma (19%) [5]. Similarly, in 67% of our cases, the epithelioid component was squamous cell carcinoma, and it was adenocarcinoma in 33%. The sarcomatous component involves poorly differentiated osteosarcoma, chondrosarcoma, or rhabdomyosarcoma [5, 7, 8]. In our cases, osteosarcoma, chondrosarcoma, and spindle cell carcinoma were mostly seen in the sarcomatous component.

Interestingly, one of our cases had a synchronous tumour at the time of diagnosis. The pathological diagnosis of one of these two tumours was carcinosarcoma, whereas the other one was a stage Ia (T1N0M0) squamous cell carcinoma located in the other lobe, without involvement of the shared lymph nodes. Synchronous tumours are rare [14], and synchronous tumours in which one component is carcinosarcoma are rarer, with no other case reported in the English-language literature.

Complete surgical removal of the tumour with negative tumour margins constitutes the desired treatment approach. There is limited information on systemic treatment options such as chemotherapy and radiotherapy [15, 16]. Nevertheless, the aggressive nature and poor differentiation of this tumour render the treatment difficult and result in a poor prognosis [17]. Although relatively satisfying survival rates have been reported in some series (49.3% by Petrov et al. and 57% by Yazici et al.), the survival was poor in others (21.3% in Koss et al.) [5, 12, 18]. In a most recent series involving sarcomatoid carcinoma reported by Park et al., survival rates have been reported as 54.3% and mean followup was 16.07 months [13]. In our series, the median survival time was 9 months.

Although complete resection was performed on all of our tumours, the survival time was short, as in Koss et al. [5]. Fishback et al. reported that a tumour diameter larger than 5 cm, disease stage higher than stage I, and the presence of lymph node involvement had negative effects on the prognosis [4, 19].

In most cases, the preoperative diagnosis is incomplete, and lung resection is needed for a definite diagnosis. The complete, correct diagnosis in five of our six cases could not be made before resection. Although local metastases after complete surgical resection are rare in NSCLC, we recorded local metastases in two of the PCS cases. We explained this as due to the aggressive local behaviour of the tumour, as did Sato et al. [20].

Metastasis is frequent and is most common to the lymph nodes, followed by the kidneys, bones, liver, and brain [5]. One of our cases died of brain metastasis at 10 months.

In conclusion, a preoperative diagnosis of carcinosarcoma of the lung is difficult due to the composition of the different histopathological structures. This tumour can present as a solitary pulmonary nodule or as a component of synchronous tumours. Unlike reports in the literature, it can also involve the lower lobe. Complete surgical resection is still the only effective treatment for pulmonary carcinosarcoma, but the prognosis is poor. The clinical and prognostic properties of carcinosarcomas are still unknown, and more studies and larger multicentric series are needed.

## Figures and Tables

**Table 1:** Characteristics of the patients.

Patient	Age	Cigar (pack years)	Symptoms	Endobronchial Component	Localization	Diameter (cm) CT/surgical	Preoperative diagnosis
1	63	90	Cough hemoptysis	Yes	Right lower lobe	3/3	NSCLC (epidermoid)

2	66	40	Chest pain, Hemoptysis Cough	Yes	Right central	8/10	NSCLC with neuroendocrine differantiation

3	54	60	Hemoptysis Chest pain Cough	No	Right hiler intermediate Bronch	5/6,5	Carcinosarcoma

4	53	50	Hemoptysis Chest pain Cough	No	Left lower lobe	10/14,5	NSCLC

5	55	30	—	No	Left upper lobe	3/3,7	None

6	56	40	Chest pain Dyspnea	No	Left lower lobe	3,7/5,5	NSCLC

NSCLC: Non-small-cell lung carcinoma.

**Table 2:** Diagnostic characteristics of the lesions.

Patient	TNM	Stage	Cellular components of end diagnosis	Resection	Followup time (m)	Prognosis
1	T3N0M0	IIB	Squamous Chondrosarcoma	Right Lower lobectomy	10	Death

2	T3 N1M0	IIIA	Squamous Osteosarcoma	Right extrapleural Pneumonectomy	9	Death

3	T3N1M0	IIIA	Squamous Spindle cell	Right lower sleeve bilobectomy	25	Alive

4	T3N0M0	IIB	Adenocarcinoma Osteosarcoma	Left lower lobectomy	5	Death

5	T2AN0M0	IB	Adenocarcinoma Chondrosarcoma	Left upper lobectomy	6	Alive

6	T2BNOMO	IIA	Squamous Osteosarcoma	Left lober lowectomy	3	Death
